# The Resurgence of Standing in Judicial Review

**DOI:** 10.1093/ojls/gqae005

**Published:** 2024-03-14

**Authors:** Joanna Bell

**Keywords:** judicial review, administrative law, standing, *locus standi*, planning, parole, procurement

## Abstract

It is now commonplace for courts to remark that standing to seek judicial review is ‘context-sensitive’. The questions of how the courts adapt standing to context, and whether they do so appropriately, have, however, received remarkably little scholarly and judicial attention. This is perhaps because, until recently, there has been relatively little in the case law to spark scholarly interest. Standing, however, is in the midst of a resurgence. This article makes use of a distinction between three types of judicial review case—challenges to (i) favourable targeted, (ii) unfavourable targeted and (iii) non-targeted decisions—as a mode through which to explore the growing body of standing case law. In doing so, it both seeks to further understanding of how courts determine what constitutes a ‘sufficient interest’ and to highlight areas of the law in need of clarification or reconsideration.

## 1. Introduction

In a now oft-cited passage in *AXA General Insurance Ltd v Lord Advocate*, Lord Reed remarked that ‘a requirement that the applicant demonstrate an interest in the matter complained of will not … operate satisfactorily if it is applied in the same way in all contexts’.^1^ His Lordship went on to say that ‘what is to be regarded as [a] sufficient interest … depends therefore upon the context, and in particular upon what will best serve the purposes of judicial review in that context’.^2^ The question of what constitutes standing to bring judicial review does not, in other words, admit of a single, straightforward answer. Here, as in other areas of judicial review, context is everything.^3^

Remarks such as these, which have become commonplace in judicial review case law, give rise to a series of important questions. Precisely which aspects of the context matter when a court applies the sufficient interest test, and how do they matter? More broadly, are courts adapting the standing test in a coherent and justifiable way? If the standing test is to be applied predictably and appropriately, robust answers to these questions are necessary. To date, however, they have received scant attention. A major reason is perhaps that, until recently and for various reasons,^4^ case law has been rather thin on the ground. Standing is, however, in the midst of a resurgence. Questions about the applicability of the sufficient interest test have been at the heart of a growing body of case law in recent years. These challenges provide administrative law scholars with a good deal more legal material with which to work.

This article has two main aims: first, to further understanding of how courts apply the sufficient interest test in different types of judicial review challenge; and secondly, to critique the state of the law, including identifying areas in need of reconsideration or clarification. The article advances both of these aims by drawing a distinction between three categories of judicial review case—challenges to (i) unfavourable targeted, (ii) favourable targeted and (iii) non-targeted decisions—which it uses to navigate the case law. Section 2 offers an introduction to standing and its resurgence. Section 3 introduces the distinction between the three types of case. Sections 4–6 explore how courts have resolved standing issues in each category. Section 7 offers some reflections on the bigger picture and section 8, finally, concludes.

A few upfront, clarificatory points are useful. First, the focus of the article is on standing to seek judicial review in the English and Welsh legal system and therefore primarily the ‘sufficient interest’ test. Where illuminating, however, the article engages with statutory appeals limited to ‘persons aggrieved’,^5^ which are governed by equivalent principles.^6^ Secondly, within judicial review, entitlement to rely on certain grounds is governed by different tests which fall beyond the scope of the article. This article does not, for instance, consider the ‘victim’^7^ test applicable in human rights claims, nor standing to rely on provisions of European Union law.^8^ Thirdly, this article does not discuss everything of interest in recent case law on standing. For example, the courts have recently revisited the capacity of unincorporated association to bring proceedings.^9^ This matter is important, but distinct from the central questions of this article.

Finally, this article unashamedly focuses primarily on case law which is not well known. Correlatively, case law which is well known will not be a prominent feature of discussion. Both are inevitable consequences of the nature of the project. Standing issues are often determined at the High Court level, sometimes exclusively at the permission stage, and are not often appealed. Rigorous doctrinal study of standing therefore requires working primarily with first instance, low-profile decisions.^10^ Meanwhile, cases such as *Fleet Street Casuals*,^11^*Greenpeace*,^12^*Rees-Mogg*^13^ and *World Development Movement*,^14^ which have featured prominently in the collective ‘administrative law imagination’,^15^ remain important, and their major themes continue to have resonance.^16^ They are, however, the start of the story of how the courts have made sense of the sufficient interest test, not the end.

## 2. Standing and Its Resurgence

In order to demonstrate standing in judicial review, an applicant must show a ‘sufficient interest’ in the matter to which their application relates. This formulation was incorporated into primary legislation in 1981^17^ on the recommendation of the Law Commission.^18^ The Law Commission recommended the phrase to afford courts flexibility to ‘further develop the requirement of standing … having regard to the relief which is sought’.^19^ Its view, in line with many of its consultees, was that a more precise definition would unduly constrain.

Tasked with interpreting the sufficient interest test, the courts have similarly avoided the ‘risk’ of attempting to define more precisely ‘what constitutes standing in the public law context’.^20^ Instead, they have tended to highlight general themes and to emphasise the test’s context-specific nature. In terms of general themes, the most prominent have been the importance of a broadly ‘liberal’^21^ approach which enables the rule of law to be upheld^22^ and the inappropriateness of equating standing with a legal right or financial stake.^23^ At the same time, courts frequently emphasise that the proper approach to standing turns acutely ‘both on the statutory context and the factual context’^24^ of a case. The concept of a sufficient interest has been described as not ‘lend[ing] itself to exhaustive definition … [and] inherently elastic depending on the particular context and circumstances’.^25^ While, therefore, ‘there are situations where the court adopts a very liberal approach to the issue of standing’, this is not universally true.^26^

The desire to preserve flexibility is understandable. Judicial review functions as a ‘procedural mop’.^27^ As a ‘remedy of last resort’,^28^ its role is to absorb arguable legal challenges which are not the subject of statutory rights of appeal. The result is a deeply varied body of case law. However, without more, knowing that standing is context-sensitive is not particularly helpful. There is a need for careful study of precisely *how* standing is adapted to different types of judicial review case. Despite its importance, this issue has received surprisingly little scholarly engagement. There was a boom in standing literature in the 1990s and early 2000s. The focus, however, tended to be on the standing of campaign groups, an issue connected with broader themes concerning group litigation,^29^ and constitutional theory.^30^ More recently, standing has largely fallen off the academic radar.^31^

A probable reason is that there has been little to excite academic interest in the subject. The UK Supreme Court has engaged with the sufficient interest test only twice, and in both cases indirectly.^32^ At the Court of Appeal and High Court levels, standing case law has been somewhat sparse and sporadic.^33^ Some, furthermore, have perhaps assumed that the emphasis in early case law on the importance of a ‘liberal’ approach rendered standing essentially a non-issue.^34^

Recently, however, standing has been experiencing a resurgence. While the current informational landscape on judicial review precludes a comprehensive quantitative study,^35^ public authorities appear to be contesting applicants’ standing with greater frequency than in the past. Reasoning on standing is, furthermore, often going well beyond a few cursory judicial remarks^36^ and increasingly occupying substantial portions of court judgments.^37^

This resurgence is explained by a number of developments. The remarks of the Independent Review of Administrative Law in 2020 that it is ‘open to defendants in judicial review proceedings to do more to challenge the standing of claimants … than they perhaps do at the moment’^38^ has perhaps influenced practice. Schiemann LJ once remarked extrajudicially that counsel’s willingness to contest standing is a matter of fashion^39^ and it appears that (contesting) standing is back in vogue. The rise of crowdfunding,^40^ especially by organisations, is also leading to more in the way of boundary-pushing case law. Indeed, the controversial public interest group the Good Law Project (GLP), which brings ‘campaigning litigation’^41^ in pursuit of its broad mission to use ‘the law to hold power to the account, protect the environment and ensure no one is left behind’,^42^ has been generating its own microcosm of standing case law, much of which will be discussed below. Whatever the reasons, standing issues have been at the heart of case law with increased frequency in recent years. This development both aids and emphasises the need for serious doctrinal study of how standing is adapted to context.

## 3. Three Categories of Case

Exploring the adaptation of standing requires the selection of a method. A possible approach, for instance, could be to identify a list of considerations courts have relied on when applying the sufficient interest test: the nature of the applicant’s interest, their expertise and experience, the existence of alternative challengers, etc. This has been a common approach in both textbooks^43^ and case law.^44^ However, it has clear drawbacks. Most notably, knowing that courts have relied on these factors does not reveal much about whether they matter differently, in different types of case. This article makes use of a different and, it is argued, more illuminating approach. It organises the case law into three broad categories—challenges to (i) unfavourable targeted, (ii) favourable targeted and (iii) non-targeted decisions—and explores how the courts have approached standing in each. There are two main benefits to this approach. First, this system provides a helpful map through the case law, grouping together cases which share important similarities, and illuminating patterns which would not otherwise be visible. Secondly, is also highlights the distinct normative issues to which these different kinds of case give rise.

Turning to the details of the trichotomy, the first and second categories comprise challenges to decisions which are *targeted*. For present purposes, ‘targeted’ decisions confer benefits or place burdens on individuals. They are, in other words, decisions *about* a specific person or organisation. A large portion of administrative decision making in England and Wales entails the application of legal standards to individuals, often who have applied for consideration.^45^ Indeed, the bulk of judicial review case law concerns challenges to decisions of this kind.^46^ The category includes, for instance, many decisions in the immigration, social security, prisons, planning and licensing contexts.

Targeted decisions may be ‘about’ individuals and organisations in different ways. Most obviously, many targeted decisions, such as those in the immigration and housing contexts, concern *personal* eligibility for a form of treatment. However, some decisions properly classified as ‘targeted’ are ‘about’ individuals or organisations in a different sense. Consider a grant of planning permission. Planning permission attaches to land, meaning that a person who subsequently acquires a proprietary interest may benefit from an unexpired permission. A grant of planning permission is not, therefore, *personally* about any specific individual. It is, however, targeted in the sense that it concerns the ability of whichever person(s) hold a relevant interest to develop land.

The first category comprises targeted decisions which are ‘unfavourable’ and the second those which are ‘favourable’. A targeted decision is unfavourable, broadly, if its effect is to refuse a benefit or to subject an individual to a (continued) burden. A favourable decision, in contrast, confers a benefit or removes or reduces a burden. A refusal to direct the release of a prisoner by the Parole Board is a good example of an unfavourable targeted decision: its effect is to continue a restriction on liberty. In contrast, a decision to grant a licence confers a benefit on an individual as is therefore favourable.^47^ Usually, targeted decisions are straightforwardly classified as favourable or unfavourable. In unusual cases, however, parties may have different ways of seeing things. A decision by a professional disciplinary tribunal to impose suspension will usually be seen as unfavourable by its subject. A regulator, however, may see the sanction as favourable due to being unduly lenient.^48^ In unusual cases of this kind, the discussion below assumes the viewpoint of the applicant for judicial review.

A third and final category of case comprises challenges to non-targeted decisions. These are decisions which lack a particularised focus and are not ‘about’ any one individual. Non-targeted decisions have a more abstracted quality, often establishing general rules, principles or policies to govern a common type of situation. Abstract*ed*, rather than abstract, is the appropriate descriptor. Non-targeted decisions do not have an individual subject: they are abstract*ed* from a particular applicant or set of facts. They can, however, be more or less abstract, in terms of the level of precision in which they are formulated. The vast majority of delegated legislation^49^ falls within this category, as do most non-legislative rules, guidance documents and policies. The category also includes decisions which set a general policy direction, such as the decision to begin the UK’s process of leaving the European Union^50^ or the decision to pursue restructuring of the National Health Service.^51^

Some final points of clarification should be noted. First, the term ‘decision’ is used throughout the article in a broad sense to encompass any practice, ‘enactment … decision, action or failure to act’^52^ which is amenable to judicial review. Secondly, it is, of course, possible for applicants in judicial review to challenge multiple decisions falling within different categories, as where an applicant challenges both a targeted decision and the legal rule under which it was made. Thirdly, while the vast majority of judicial review cases fall very neatly into one or more of the above categories, the trichotomy does not accommodate every possible kind of challenge which might be brought via judicial review. For instance, cases where applicants do not challenge a decision but seek an advisory declaration on the content of the law^53^ do not fall into any of the categories. Finally, in some places, it is useful to make further distinctions within a category. As will be seen below, this is especially true of challenges to favourable targeted decisions (where it is helpful to examine individually the approach taken in planning, parole and procurement cases). The threefold taxonomy alone, in other words, does not illuminate everything of importance about the case law. It is, however, a valuable starting point.

## 4. Category 1: Unfavourable Targeted Decisions

As explained above, an unfavourable targeted decision is one which negatively impacts the interests of a person or organisation who can be regarded as its ‘subject’. Refusals of planning permission, licences, social security benefits and parole are all examples. In such cases, it is beyond doubt that the subject of the decision has a ‘sufficient interest’ to seek review.^54^ Indeed, the vast majority of challenges to decisions of this kind are, without contention, brought by their subjects.^55^ This is hardly surprising. The subject has, after all, a unique stake in the decision and its negative impact provides a motivation for challenging it.

Where contentious standing issues arise in these cases, therefore, they focus on the ability of *others* to seek review. A range of legal mechanisms exist to enable persons or organisations to act on behalf of another. Litigation friends, for instance, may be appointed where the subject of a decision is a child or a person who lacks mental capacity.^56^ This is what Peter Cane calls ‘surrogate’ standing.^57^ The issue being considered here is distinct. It is whether a person, external to a targeted decision, can seek review *in their own name*.

In cases giving rise to this question, the courts have consistently answered it negatively. The sufficient interest test has, in other words, been applied highly restrictively in this type of case. Some recent examples neatly illustrate this proposition.^58^ In *Zafar*, the applicant sought to challenge a planning enforcement notice issued in relation to his mother’s property requiring the demolition of an unpermitted extension. The High Court accepted that the applicant sought judicial review as a ‘dutiful and concerned son’ seeking to protect his unwell mother. It nonetheless held that he lacked a sufficient interest to proceed: ‘the interest in the enforcement notice [was] that of his mother … The claimant ha[d] no interest in the property and no direct interest that is not that of his mother’.^59^ Similarly, in *AB*, a teacher sought to ventilate ‘substantive safeguarding concerns’ about her employer’s treatment of a student who wished to transition genders. The High Court held that she lacked standing. She was not acting in a formal capacity as a representative of the pupil and her claim to be acting as a ‘whistleblower’ was insufficient.^60^

A similarly restrictive approach has also been taken in two recent cases in Northern Ireland. In the first, in line with earlier, similar authorities from both Northern Ireland^61^ and England and Wales,^62^ a prisoner lacked standing to challenge the remuneration levels paid to his legal representative. It was held to be clear from the case law that ‘the only potential applicant in this case [was] the applicant’s solicitor’,^63^ who was the subject of the decision. In the second, the High Court held that the only persons who have standing to challenge findings of facts relating to alleged wrongdoing by individual police officers are those officers. While the Northern Ireland Retired Police Officers’ Association had standing, as a matter of public law,^64^ to challenge the ombudsman’s general practice of making findings of corruption, it lacked standing to challenge the handling of particular allegations against individual officers.^65^

The unifying feature of all these cases is a narrow understanding of what constitutes a ‘sufficient interest’. As several of the cases illustrate, it is not enough for the applicant to convince the court that they seek review out of ‘genuine concern’^66^ with the issues. Applicants have also been found to lack standing where an unfavourable targeted decision directed to another may affect them materially. In the older case of *Connolly*, for instance, landlords lacked standing to challenge refusals to reconsider the quantum of housing benefit paid to their tenants despite the decisions’ creation of risk that the tenants would be unable to pay their rent.^67^ Rather, across these cases, a ‘sufficient interest’ is equated with *being* the subject of the unfavourable decision.

The narrowness of the courts’ approach in these cases is a striking reminder that standing is not uniformly ‘liberal’.^68^ There are also generally good reasons for the adoption of a narrow approach in cases of this kind. One explanation offered in some of the case law is that the courts refrain from undermining specific statutory procedures which enable the subject of a decision to ventilate complaints. In *Zafar*, for example, the mother had elected not to use a statutory right of appeal against the enforcement notice. The High Court remarked that her son ought not to be permitted to ‘get around’ his mother’s inaction ‘by trying to advance [through judicial review] precisely the same matters that would be relevant to such an appeal’.^69^ That Parliament has created a route for challenging a decision, however, should not be treated as determinative of the question of whether a third party has a sufficient interest to seek judicial review. Such a position would undermine the well-established principle that parliamentary ouster of ‘the court’s jurisdiction by way of judicial review requires clear and explicit language’.^70^

The better justification for the narrowness of the courts’ approach has two components. First, permitting an external person or organisation to challenge an unfavourable targeted decision has a significant practical impact on the decision’s subject. For instance, in *AB*, one reason the High Court declined to recognise the applicant as having standing was the significant ‘intrusion into [the subject’s] right to respect for private life that would inevitably be involved by the deployment of evidence’^71^ about them. Recognising the standing of third parties would also create a practical dilemma for the decision’s subject. In a case, for instance, where a third party sought to challenge a refusal of planning permission, the developer would face a choice between two options: on the one hand, participating in the challenge as an interested party,^72^ either appearing as a litigant in person or funding legal representation,^73^ and exposing themselves to the risk of an adverse costs order;^74^ and on the other hand, being unrepresented in a legal dispute in which their interests are uniquely at stake. Secondly, unlike favourable targeted decisions,^75^ that impact cannot, absent something highly unusual, be justified by the need to enable others to proceed to provide a check against legal error. Because of the unfavourable nature of the decision, the subject will usually have an incentive to challenge the decision and will be a more suitable ‘alternative challenger’^76^ than an external party.

There are some possible, but ultimately unconvincing, objections. It might be argued, for instance, that the courts’ approach would prevent the possibility of a well-resourced applicant being able to challenge a decision where its target could not afford the notorious financial expense of judicial review.^77^ In such a scenario, however, the claim would be initiated by the subject with the financial support of the external party. An objector might also point out that the narrow approach would prevent a third party from challenging an unfavourable targeted decision for the purpose of obtaining clarification on a point of law in which they have a direct interest. While this might be seen as reducing the courts’ ability to perform its legal ‘expository’ function,^78^ it is difficult to see why the decision’s subject should be exposed to the practical impacts of unwanted litigation outlined above for the third party’s benefit.

It should finally be noted that the courts have, rightly, departed from the full strictures of their usual narrow approach in exceptional cases. For instance, *Islam* concerned a challenge by a father to the revocation of his son’s citizenship.^79^ Notice of the decision was served at his son’s last known address in Bangladesh.^80^ However, his son was at the time detained in a non-state armed group facility in Syria. The High Court remarked that the son, as subject of the decision, was the ‘obvious claimant’^81^ but, in the circumstances, regarded the father as having a sufficient interest. This is an entirely justifiable decision, readily explainable by the high degree of unlikelihood that the son would learn of the decision in time to seek review.

## 5. Category 2: Favourable Targeted Decisions

Challenges to favourable targeted decisions are similar to, but also importantly different from, the cases within the first category. They are similar in the sense that they have a ‘subject’. As in the first category, permitting a third party to seek review of decisions of this kind will render that subject vulnerable to a range of negative practical implications. Indeed, given the favourable nature of the decision, the practical implications will often be more severe because the subject stands to lose, rather than gain, something. Consider a decision by the Parole Board to release a prisoner. Permitting a third party to seek review would have at least the following implications for the prisoner: the possibility that the High Court may make an interim order staying release;^82^ the possibility that sensitive documents containing personal information will be made available to the challenger and the public more broadly;^83^ facing the choice of appearing as an interested party (either in person or by funding legal representation) or being unrepresented in a hearing; the prospect of the release decision being quashed; and the uncertainty, stress and emotional anguish which would undoubtedly flow from the above.

However, unlike unfavourable targeted decisions, the subject of a favourable decision, as its *beneficiary*, will, absent something highly unusual, have no incentive to challenge it, no matter how egregiously tainted by legal error. This creates issues from a rule-of-law standpoint. As Macur LJ and Chamberlain J cogently explained in *McCourt*:

The main task of the courts in the field of public law is to ensure that those exercising public power do not overstep the legal limits on their powers. But the courts can only perform that task if arguably unlawful exercises of public power are brought before them. Their ability to preserve and vindicate the rule of law depends on *the existence of effective mechanisms which enable that to happen*.^84^

Challenges to favourable targeted decisions, in other words, give rise to some of the most difficult standing issues because they require the courts to strike a difficult balance between competing considerations: on the one hand, protecting the subject of the decision against a range of material and emotional impacts; on the other, ensuring that judicial review is able to act as an ‘effective mechanism’ for scrutinising arguable legal errors.

The courts have been required to strike this balance in a number of different administrative decision-making contexts.^85^ Three have, however, been particularly prevalent in the case law: planning, parole and procurement. This section begins by discussing each body of case law in turn. It then explores the possibility of developing a method for determining what constitutes a sufficient interest to challenge other categories of favourable targeted decision.

### A. Planning

Since 1947, a person intending to develop land is required to obtain planning permission.^86^ The responsibility of deciding applications for permission rests primarily on local planning authorities. However, the Secretary of State also has the power (often delegated to the planning inspectorate) to call in^87^ and hear appeals.^88^ Howsoever decisions are taken, wide statutory participation rights are accorded to anyone who wishes to speak in favour of, or against, proposed development. Local authorities, for instance, are subject to obligations to publicise planning applications and to invite representations from any person who wishes to make them.^89^ Where a decision is appealed, representees are informed and invited to make further comments for consideration.^90^

Final decisions on planning applications are challengeable either by judicial review or by ‘persons aggrieved’ through a statutory appeal to the High Court.^91^ In both instances, when determining whether a person has standing, the courts have treated the extent of the applicant’s participation at earlier stages of the decision-making process as the primary factor.^92^ ‘Any person who has attended and made representations at [an] inquiry’ (or other relevant procedure) is usually regarded as having ‘the right to establish in the courts that the decision is bad in law’.^93^ In contrast, that an applicant ‘has failed to state his objection at the appropriate stage of the procedure’^94^ is usually regarded as a basis for denying standing. Exceptions have been recognised for unusual cases. For instance, where ‘the inadequacy of the description of the development … could well have misled the appellant or put him off his guard’,^95^ lack of participation will not negate standing.^96^ In general terms, however, failure to ‘avail [oneself] of the opportunities which Parliament has afforded for participation in the process’^97^ will prevent an applicant from proceeding.

In the context of challenges to grants of planning permission, in other words, ‘sufficient interest’ and ‘person aggrieved’ have both come to acquire a specific meaning. The courts have long moved beyond the idea that standing requires a proprietary right.^98^ Standing to challenge a grant of planning permission, rather, can be *acquired* by any person who makes use of the legislative right to raise objections.

In the specific context of planning decision making, this approach to standing strikes a clear and defensible balance between the competing considerations. The participatory rights created by planning legislation are much broader than those existing in other fields and, notably, there are no legal limitations on who can take part.^99^ This enables a wide range of objections—proprietary, social, environmental, etc—to be raised. Extending standing to any person or organisation who has participated in the decision-making process thereby ensures that judicial review can act as a mechanism for upholding the wide variety of laws and policies governing planning decision making. At the same time, denial of standing to those who have not made use of earlier opportunities to voice concerns prevents unanticipated legal objections from being raised at a very late stage in proceedings. This provides an important safeguard against delay and unforeseen expense for developers and others (including public authorities) with an interest in development.

### B. Parole

The lines of standing have been drawn very differently in cases concerning Parole Board decision-making on the potential release of a prisoner. Decisions to refuse to direct release, as unfavourable targeted decisions,^100^ can, of course, be challenged by the prisoner. In the case of decisions to direct release (favourable targeted decisions), two actors have been afforded standing: first, the Secretary of State for Justice (SSJ), who, as a party to the hearing, has long been recognised as having a sufficient interest to seek review; and secondly, victims and (where the victim is deceased) their representatives. Thus, in *DSD*, the standing of victims challenging the release of a convicted sex offender was not contested and the Divisional Court inclined to the view that the concession was rightly made.^101^ More recently, in *McCourt*, the High Court recognised the mother of a deceased victim as having a sufficient interest to seek review of the release of her daughter’s murderer.^102^

Importantly, however, the case law suggests these are the *only* categories of person with standing. Thus, in *DSD*, the Mayor of London’s concern to protect women in the city did not constitute a sufficient interest.^103^ Similarly, in *McCourt*, the Divisional Court strongly resisted the proposition that the applicant’s campaign work in the field of victim’s rights was a basis for affording her standing.^104^ Macur LJ and Chamberlain J explained:

[T]he rule of law does not require that Parole Board decisions in individual cases should be challengeable by any member of the public. In most cases, there is likely to be a small class of persons who are much more directly affected than the public at large. If no one in this class is prepared to bring a challenge, it can be properly assumed, without offending the rule of law, that there is no need for the court to entertain one.^105^

By drawing the boundaries of standing in this way, the courts rely on the SSJ and a prisoner’s past victims to act as a collective filter for drawing arguably unlawful instances of decision making to the attention of the High Court. This role is broadly analogous^106^ to that played by the Attorney General in challenging unduly lenient criminal sentences^107^ or the General Medical Council in appealing unduly lenient sanctions by the Medical Practitioners Tribunal.^108^ The practical reality will surely be that the SSJ, who has a strong political incentive to ensure that the Parole Board undertakes a rigorous assessment of the risk posed by prisoners,^109^ will play the primary role. Indeed, the recent introduction of a right to request reconsideration strengthens the SSJ’s capacity to seek redress for error.^110^ By extending standing beyond the SSJ to victims,^111^ the courts have, furthermore, provided a safety net for catching instances of arguable legal error not challenged by the SSJ. This may prove to be particularly important in cases, like *DSD*,^112^ which concern Parole Board practices widely assumed to be lawful.

There are, furthermore, good reasons for not extending the boundaries of standing beyond these groups. As explained above,^113^ permitting an external party to seek review exposes prisoners to an array of acute practical and emotional impacts. Parole and criminal justice, furthermore, are politically controversial topics and the Divisional Court in *McCourt* was evidently concerned by the prospect of prisoners being exposed to those impacts by applicants pursuing weak claims for the primary purpose of garnering publicity for a cause.^114^ Finally, the main objection to the Divisional Court’s approach is that widening the doors of standing further would reduce the risk that arguably unlawful instances of decision making will not be brought to the attention of the High Court by either the SSJ or victims. A more effective response to this risk, however, would be to seek to improve practices within the Ministry of Justice relating to the scrutiny of Parole Board decisions.

### C. Procurement

The category of judicial review challenge which has most vexed the courts applying the sufficient interest test encompasses challenges to the award of public contracts. For many years, the procurement process has been governed by various iterations of the EU law-derived Public Contracts Regulations (PCRs).^115^ These regulations have now been replaced by the Procurement Act 2023. The 2023 Act, however, has not substantially altered the features of the regulations described below. The procurement regime will therefore inevitably continue to give rise to similar questions concerning standing, meaning the pre-Act case law will remain relevant.

The procurement regime requires the award of public contracts worth more than qualifying threshold amounts^116^ to be carried out through one of a range of specified procedures.^117^ Procurement processes are also governed by general principles laid down in the regulations/statute^118^ as well as the principles of judicial review.^119^ Importantly, specific legislative provision is made for enforcement. Public authorities are placed under a duty to comply with the provisions of the legislative regime, and that duty is specified to be ‘owed to’ economic operators.^120^ Where these duties are breached, economic operators who suffer, or risk suffering, loss or damage are empowered to bring proceedings in the High Court.^121^

The only recourse for parties other than economic competitors, in contrast, is to apply for judicial review, giving rise to the question of who had a ‘sufficient interest’ to do so. It is now well established that the legislative enforcement mechanisms are not exhaustive, and that judicial review may sometimes be available.^122^ Beyond this, however, the case law is in a highly unsatisfactory state.

*Chandler* ^123^ is widely regarded as the leading case. The challenge concerned a challenge to an agreement between the UK government and University College London relating to an academy school.^124^ The Court of Appeal held that the applicant lacked standing on the basis that she sought review primarily out of ideological opposition to academy schools. Importantly, however, the court also offered obiter reflections on what constitutes a ‘sufficient interest’ to challenge the award of a contract more generally. The case has commonly been understood as identifying two scenarios in which a non-economic operator will have standing: first, where the applicant is ‘affected in some identifiable way’ by the purported unlawfulness, such as where use of a lawful process ‘might have led to a different outcome that would have had a direct impact on him’; and secondly, where ‘the gravity of a departure from public law obligations … justif[ies] the grant of a public law remedy in any event’.^125^

The boundaries of standing drawn in this case are of a different type to those crafted elsewhere. In planning and parole, the courts have identified clearly defined groups—participants in consultation processes (planning), the SSJ and victims (parole)—who are afforded standing.^126^ The borders of the two scenarios drawn in *Chandler* are much less precise and have given rise to considerable legal complexity.

*Chandler*’s first category focuses attention on the extent to which the applicant is ‘affected’ by the alleged legal error. One source of disagreement^127^ in the post-*Chandler* case law has concerned whether it is necessary for an applicant to show that a lawful process might have made a difference to the outcome of the procurement process. Dove J in *Wylde*^128^ considered that it was. More recently, Chamberlain J took the opposite view.^129^ Chamberlain J’s view is more consistent with the Court of Appeal’s reasoning in *Chandler* and minimises the practical difficulties of meaningfully assessing the likelihood that a legal error made a difference.^130^ Even with this ambiguity settled, however, the courts have not adopted a clear and consistent approach to determining whether an applicant is ‘affected’. In some cases, for instance, it has been accepted without argument that potential users of the service or goods have standing.^131^ In contrast, in *Chandler*, the Court of Appeal specifically remarked that the fact that the applicant had school-age children in the area of the proposed school was insufficient.^132^

Furthermore, the Court of Appeal in *Chandler* did not articulate a clear and convincing rationale for treating the extent to which a person is ‘affected’ by a decision as a point of delineation between those who do and those who do not have standing. It is questionable whether there is such a rationale. A regular supplier of an economic operator which misses out on a contract, for instance, may be able to demonstrate that their financial interests are ‘affected’.^133^ It is not obvious, however, why this impact should entitle them to bring a claim where the standing of others is denied.

The second route identified in *Chandler* enables an applicant to establish standing by pointing to especially ‘grave’ instances of alleged unlawfulness. An obvious issue with this category is the conflation of the requirement to establish standing with the merits of a challenge. Elsewhere in its judgment, the Court of Appeal in *Chandler* remarked that ‘it would drive a coach and horses through the requirement for standing if the importance of the issue justified standing’,^134^ and yet this is precisely how the second route operates.

More importantly, the metrics by which a court is supposed to judge ‘gravity’ are far from clear. The Court of Appeal primarily seems to have had in mind a focus on the seriousness of the alleged breach, rather than the likelihood of success. But what factors are relevant in assessing gravity in this sense? The extent to which the public is aware of the issue, meaning the breach threatens to damage public trust? The degree of awareness on the public authority’s part that it may have been breaching the law? The amount of public funds spent on the contract? The extent to which the legal question(s) in issues may be relevant in other cases? The Court of Appeal judgment offers no guidance. It is therefore unsurprising that judicial assessment of gravity in the post-*Chandler* case law has been characterised by inconsistency. In two similar recent challenges brought by the GLP to the award of contracts during the coronavirus pandemic, for instance, the judges took opposing views on whether the GLP had standing on this second basis.^135^

The Court of Appeal has recently indicated that it has the appetite to revisit *Chandler*. In *GLP, Public First*,^136^ the High Court judge, applying *Chandler*, had accepted the GLP had standing. Burnett LJ expressed surprise that this finding had not been appealed, remarking:

[T]he judge’s decision … was based so far as concerns the Regulations on the obiter dicta of this court in *Chandler* … The question of standing for complete strangers to the procurement process with no commercial interests both under the Regulations and on public law grounds is a question ripe for review.^137^

The criticisms above certainly afford reasons to depart from *Chandler*. What Burnett LJ appears to have in mind as a replacement, however, is two distinct approaches to standing depending on whether the applicant is seeking to rely on provisions in the PCRs (or now the Procurement Act 2023) contrasted with the grounds of review. This would be a problematic route to take, not least because provisions within the Procurement Act overlap considerably with general public law principles.^138^

### D. Drawing the Boundaries of Standing in Challenges to Favourable Targeted Decision: A Possible Method

At this juncture, it is useful to attempt to tie together some of the threads from the earlier discussion by considering the following question: where a court is faced with a challenge to a favourable targeted decision, how should they identify the boundaries of standing? Several important lessons emerge from the discussion above. One is that an appropriate response may be to limit standing to an identifiable group or groups. This has been done to good effect in planning and parole. Another, however, is that there is not a singular, appropriate way of drawing the boundaries of standing across all types of challenge to favourable targeted decisions. It was explained above, for instance, that in planning cases the court has assimilated standing with participation in the decision-making process. This approach is defensible in the planning context because of an unusual feature of the statutory framework: legally unlimited participation rights. This feature, however, is not replicated in other areas of administrative decision making, including parole^139^ and procurement,^140^ and would result in an unjustifiably narrow approach to standing if used there. Challenges to favourable targeted decisions, in other words, cannot adequately be dealt with by a singular ‘test’ of standing. What may be needed, rather, is a *method* through which courts can identify an understanding of a ‘sufficient interest’ which is workable for a particular category of case.

An important starting point in the creation of such a method is the Divisional Court’s emphasis in *McCourt* on the need to ensure that judicial review provides an ‘effective mechanism’ for scrutinising arguable legal errors.^141^ Public authorities can make a very wide range of legal errors in the course of decision making. Different legislative frameworks create different legal demands. Some, such as the requirements to comply with Convention rights^142^ and consider the equalities implications of decisions,^143^ are generally applicable. Others are highly specific. Public authority decision making is also structured by a range of different general principles of review.^144^

These wide-ranging legal requirements promote and protect a wide range of purposes, values and interests.^145^ However, they have a common feature: namely, that if they are to be practically meaningful, there must be a legal process whereby a court can identify and remedy instances in which they are transgressed.^146^ In many areas of decision making, judicial review is the only such process available.^147^ The High Court, however, has no initiative to identify arguable legal errors for itself. The capacity of judicial review to address any given legal error is therefore wholly dependent on an applicant drawing that error to its attention. It is for this reason that standing is intimately connected with judicial review’s effectiveness as a check on legal errors. If the boundaries of standing are drawn in ways which afford access only to persons who cannot realistically be expected to challenge a decision at all, or only on certain grounds, judicial review’s effectiveness is decreased.

Turning to favourable targeted decisions, all of this explains why it would be wholly inappropriate to confine standing to the subject: beneficiaries of decisions cannot simply be expected to challenge them, no matter how egregiously tainted by legal error. The boundaries of standing must be drawn in a way that ensures that there is a meaningful prospect that the various types of legal error which might taint decision making will be brought before the court.

In crafting such an approach, a court could begin from the default position that the proper approach is a ‘liberal’ one in which standing is extended to any party able to mount an arguable challenge.^148^ The court could, however, depart from the default position if able to identify a more appropriate way of striking the balance between the interests of the decision’s subject and the imperative of ensuring judicial review remains an ‘effective mechanism’ for scrutinising arguable unlawfulness.

In working out whether such an approach exists, it would be important for courts to thoroughly probe two issues. The first issue is the extent to which the potential ill effects of an unrestricted approach to standing on the subject offer a strong reason for narrowing standing. This would require a consideration of several factors. Most importantly, it would be necessary to understand the position that permitting an external party to seek review of the decision would place the subject in. What financial and practical difficulties, for instance, does judicial review create for a developer hoping to act on a grant of planning permission? Where does review by an external party leave a prisoner? Consideration should also be given to the extent to which features of the judicial review procedure *other* than standing already provide adequate protection to the subject’s interests, including the shortened time limits which apply in some categories of judicial review challenge.^149^

Secondly, standing should only be narrowed to a group if that group has two characteristics: it is *clearly identifiable* and *sufficiently motivated*. The post-*Chandler* case law is a clear illustration of the importance of the lines of standing being defined with precision. Experience shows that broad formulations such as that the applicant must show they are appropriately ‘affected’ by a decision give rise to uncertainty. This has ill effects both for the subject of a decision and potential challengers. For the subject, it means that standing issues often cannot be resolved at the permission stage, exacerbating delays and potential legal costs. For potential challengers and their legal advisors, it creates uncertainty concerning whose name a challenge could plausibly be brought in.

The group must also be sufficiently motivated to draw the full range of possible legal defects to the attention of the High Court. Confining standing to a group which can be expected to raise only a small subset of the legal errors which might taint decision making would undermine judicial review’s capacity to act as an ‘effective mechanism’^150^ for scrutinising legal error. Consider again, by way of example, challenges to grants of planning permission. Planning decision making is structured by a wide variety of laws and policies which protect a range of different interests. To confine standing to only those complaining of an interference with a proprietary right, as the court did in the older case of *Gregory*,^151^ would render many of these laws effectively unenforceable. Proprietary right holders could be expected to challenge aspects of development with a bearing on those rights, meaning there would be no obvious route through which alleged breaches of more public-facing statutory duties (such as those to conduct an environmental impact assessment^152^ or to take account of the potential equalities impacts^153^ of development) could be raised. The more modern approach in planning, whereby standing is acquainted with participation, enables a much broader range of objectors to raise a wider set of legal concerns.

## 6. Category 3: Non-targeted Decisions

The third and final category of case falling for consideration concerns challenges to non-targeted decisions. As explained above, non-targeted decisions do not have a ‘subject’. They are generalised in their effect, often laying down standards to govern a type of situation. Challenges to statutory instruments, policies and practices^154^ often fall within this category, as do decisions setting a broad direction for future policy making.

Challenges to non-targeted decisions raise different issues to those in earlier categories. Consider again the parole context. As was seen above, in a challenge to a targeted decision to direct the release of a prisoner, the individual prisoner’s interests are uniquely at stake. Challenges to general measures such as the Parole Board Rules or policies,^155^ in contrast, are factually different. There is no one prisoner whose interests are uniquely affected by the decision. Determining challenges of this kind does not require the court to engage in detailed examination of facts pertaining to a specific individual. There is not a ‘subject’ of the decision whose interests are put on hold, pending resolution of the issues.^156^ In light of this, courts have generally taken a more ‘liberal’^157^ stance in cases concerning non-targeted decisions. A ‘generous approach’^158^ has been adopted in challenges brought by organisations and individuals^159^ alike.^160^ It is worth considering each in turn.

### A. Organisations

There are two distinct ways organisations have commonly been able to establish a sufficient interest to challenge non-targeted decisions. These routes broadly correspond to Cane’s distinction between ‘associational’ and ‘public interest’ standing.^161^ In the first, standing is established by an organisation pointing to a portion of its membership whose interests are or may be personally impacted by the measure.^162^ In the recent Northern Irish case *JR 2016*, for instance, the Northern Ireland Retired Police Officers’ Association (NIRPOA) was held to have standing to challenge a general practice by the Police Ombudsman of making findings of collusion. As a ‘representative organisation’, the NIRPOA had a sufficient interest ‘in ensuring that the statutory scheme is properly applied … since that will or may affect a wide range of their members’.^163^ Similarly, a trade union was held in *Adiatu* to have standing to argue that the Coronavirus Job Retention Scheme discriminated against women. The union had ‘5,000 members, and it [was] safe to assume that, amongst them, there will be women who are in employment’.^164^

Writing in 1995, Cane argued that a prerequisite of associational standing should be a requirement to demonstrate a ‘democratic nexus’^165^ between the organisation and its members. In Cane’s view, ‘a litigant who claims to represent the interests of identifiable individuals cannot do so convincingly unless there is a reasonably effective mechanism by which the representative can ascertain what the represented believe their interests to be’.^166^ Nearly 20 years on, the courts have not introduced such a requirement. Rather, it has generally been enough for an organisation to *have* members who are or may be adversely affected. A possible explanation is that the courts have embraced a different conception of the purpose of associational standing. Cane saw the main objects of associational standing as being to ‘facilitate access to justice by making it easier for groups … to invoke the judicial process, and to promote the efficient conduct of litigation by allowing numerous bilateral disputes … to be resolved in one set of proceedings’. On this view, associational standing enables organisations to act as vehicles through which individual members can collectively express similar legal grievances. In cases like *Adiatu*, in contrast, organisations are playing a different role: namely, one of proactively protecting the interests of their members against an arguably unlawful measure of which they may not be aware.

A second common route by which organisations establish standing is by pointing to an alignment between the legal challenge and an objective it is set up to pursue. As Cane emphasises, this type of ‘public interest’ standing is conceptually distinct from ‘associational’ standing. The organisation’s claim to a sufficient interest is not grounded in concern for the material interests of members but for a broader value the organisation exists to promote or defend. In the well-known *Greenpeace* case, for instance, Greenpeace was held to have standing^167^ as a ‘campaigning organisation which has as its prime object the protection of the natural environment’.^168^ More recently, in a challenge to a general practice of not promptly publishing contract notices during the coronavirus pandemic (*GLP, transparency*), the Good Law Project was held to have standing as a not-for-profit company with a ‘sincere interest, and some expertise, in scrutinising government conduct in this area’.^169^

While there are repeated references in the case law to the importance of organisations having ‘expertise’^170^ and ‘experience’,^171^ ‘newly established campaigning organisations’ have been assumed to have a sufficient interest.^172^ There is also no bar on organisations which define their aims specifically in terms of the pursuit of litigation. On the contrary, the objectives of some of the most prolific applicants in environmental law litigation are defined in such a way. ClientEarth, which has brought an array of high-profile judicial review challenges concerning air quality^173^ and climate change,^174^ among other matters, defines its core aim as being to ‘use the power of the law to protect all life on Earth’.^175^ On a smaller scale, challenges to grant of planning permission are often brought by local groups established for the specific purpose of resisting development.^176^

The recent case *GLP & Runnymede* may, however, come to be understood as identifying an important limit on the ability of organisations to establish public interest standing. The case concerned a challenge to a series of appointment decisions during to the coronavirus pandemic.^177^ The Divisional Court concluded that the GLP lacked standing to challenge any of the decisions, on any of its grounds.

One reading of the case is that the GLP lacked standing because of the way its objects are framed. Singh LJ and Swift J remarked that in cases in which organisations have been assumed to have standing, they have generally had a ‘particular interest and in a sense [been] representative of an identifiable group in society which was affected by the decision or policy’. *World Development Movement*^178^ and the *Refugee Legal Centre*^179^ were offered as examples. The remit of the GLP, in contrast, is framed unusually broadly.^180^ The company’s objects refer, among other things, to a concern with good administration, democracy, social justice, fairness and the environment.^181^ In the view of the Divisional Court, an organisation must not be allowed ‘in effect [to] confer standing upon itself by drafting its objects clause so widely that just about any conceivable public law error … falls within its remit’.^182^

It would be unfortunate if this case came to be understood as preventing the GLP, and organisations like it, from establishing a sufficient interest in future challenges to *non-targeted* measures.^183^ This would be inconsistent with decided case law in which the GLP has been found^184^ or assumed^185^ to have standing. It would also draw a difficult distinction between organisations which exist to protect the interests of ‘identifiable groups’^186^ (children, refugees, persons transitioning genders, etc) from those whose aims focus on more abstract values such as promoting transparency or privacy. These categories of aim are not easily distinguished, and often intertwine in practice. Furthermore, not every non-targeted decision affects a subsection of the public whose interests are safeguarded by a group.^187^

A better reading of *GLP & Runnymede*, rather, is as a reminder that in an area of judicial review where the courts have adopted a constrained approach to standing, an organisation cannot evade those limitations by relying on broadly framed objectives. *GLP & Runnymede* was fundamentally a challenge to a set of *favourable targeted* employment decisions. The Divisional Court’s view, in line with earlier case law^188^ and in a manner similar to planning and parole case law,^189^ was that, generally,^190^ standing in such cases is properly confined to individuals entitled to complain to the employment tribunals. The GLP did not fall within this category, and its broadly framed aims could not alter this. The distinction between targeted and non-targeted decisions, in other words, is crucial to properly understanding the remarks of the Divisional Court in *GLP & Runnymede*.

### B. Individuals

In cases where individuals^191^ seek to challenge non-targeted decisions, the most straightforward way for them to establish a sufficient interest is to show that they are *personally* ‘directly affected’^192^ by the measure. Courts have adopted a wide conception of what it means to be affected. For instance, in *Royal Cayman Islands Police Association*,^193^ the Privy Council overturned a decision by a lower court that several of the applicants lacked standing. The case concerned a general policy preventing mandatorily retired police officers to be re-engaged other than as a constable. The relevant applicants, unlike others, had not pursued re-engagement, meaning the policy had not been applied to their applications. The Privy Council nonetheless held that it was sufficient that the policy deterred them from seeking reappointment.

An applicant may have been directly affected by a general measure in the past.^194^ Applicants have also successfully established standing by pointing to a possible future impact. In *Adiatu*, for instance, the applicant, a ‘limb b worker’, had standing to challenge the rate of statutory sick pay paid to employees on the basis that, as he was actively seeking employment, he might acquire the status of employee and become materially affected by the rates.^195^ It is also unnecessary that an applicant show ‘special damage’^196^ above that suffered by other members of the public.^197^ Some decisions may have a material impact on all citizens, meaning that ‘any individual, simply as a citizen, will have sufficient interest … without having to demonstrate any greater impact’.^198^ For example,^199^ in *Miller*, the Divisional Court remarked that it was ‘not difficult to identify people with standing … since virtually everyone in the UK or with British citizenship will … have their legal rights affected’.^200^

Correlatively, there have been several recent cases in which individuals have been denied standing to challenge a non-targeted measure because they have not been and will not be materially affected by it. *Jones*, for instance, concerned a challenge to a decision to impose a condition under the Public Order Act 1986 requiring Extinction Rebellion protests in London to cease by a specified time. Several applicants were held to lack a sufficient interest because, unlike others, they had not been arrested for breaching the condition and there was no evidence that any ‘ha[d] been deterred from protesting’.^201^ Similarly, a doctor who had been vaccinated and did not work in a care home lacked standing to challenge regulations requiring care home operators to require workers to provide evidence of vaccinations.^202^ In *JS*, the applicant lacked standing to challenge an ‘ongoing decision’^203^ not to extend family reunion rights to child refugees as neither he nor his family had sought reunion while he was a child.^204^

In a passage which has been cited a great many times, Lord Diplock famously said in *Fleet Street Casuals* that it would be ‘a grave lacuna in our system of public law’ if a pressure groups and ‘public-spirited’ citizens were prevented from accessing judicial review.^205^ The analysis so far may suggest the courts are adopting a peculiar ‘twin track’^206^ approach which enables pressure groups to seek review for ideological reasons but denies the ‘public-spirited’ individual from doing anything other than defending their own interests.

It is important to recognise, however, that in *Jones*, *Peters* and *JS*, there were multiple applicants, some of whom were materially affected by the measure and some of whom were not. In each of these cases, the courts confined standing to the former group in an attempt to minimise the number of parties to the litigation. There are practical reasons for limiting standing in this way, which were astutely explained by the Divisional Court in *Jones*:

[A]dding unnecessary claimants is likely to increase the costs of the litigation, if only by requiring solicitors to send out extra reports on the litigation. It is also because parties to an action are in a distinct position, for example by receiving a confidential draft of the judgment.^207^

The courts are also evidently concerned to avoid the politicisation of judicial review by preventing politicians from unnecessarily being added as parties.^208^ The conclusions that applicants lacked standing in these cases, in other words, are best seen as highly circumstantial. Their interests were not sufficient *given that* other applicants also sought review. These cases, then, are illustrations that courts will prioritise applicants who have been personally impacted, not that ‘public-spirited’ citizens, who have not, are excluded.^209^

## 7. The Bigger Picture

It is useful at this juncture to tie together some of the threads of the earlier discussion. Three broad points are particularly worth making about the bigger picture.

First, briefly, the discussion throughout this article highlights very clearly that the courts’ approach to standing in England and Wales is not monolithic and is far from being ‘open’.^210^ Indeed, this article reveals a spectrum of approaches, in which the extent to which the courts’ approach to standing can properly be described as ‘liberal’ depends on the type of challenge they are faced with. In the first category of case—challenges to unfavourable targeted decisions –a highly restrictive approach has dominated. Absent something highly unusual,^211^ only one person is regarded as having a sufficient interest: namely, its subject. Meanwhile, challenges to non-targeted decisions sit at the other end of the spectrum and are characterised by a much more ‘generous approach’.^212^

Secondly, the courts have in general applied the sufficient test in justifiable ways which are responsive to the normative considerations at stake in different areas of judicial review. There are, however, a few areas in need of reconsideration or clarification. The boundaries of standing drawn in *Chandler* are vague and have given rise to a good deal of uncertainty. It is therefore to be hoped that the passage of the Procurement Act, coupled with Burnett LJ’s remarks in *GLP, Public First*, will shortly give rise to an opportunity for the courts to reconsider who has standing to challenge the award of a public contract.

More generally, appellate courts could do more in the way of providing structured guidance to the lower courts on how to determine the proper approach to standing for a particular category of judicial review case. In *AXA*, Lord Hope remarked that the Supreme Court should avoid the ‘risk [of defining] what constitutes standing in the public law context’.^213^ If his Lordship had in mind a singular definition of the term, to be applied uniformly across the full spectrum of judicial review case law, his aversion was entirely sensible. Judicial review case law is something of a ragbag.^214^ To give more precise meaning to the sufficient interest test in this way would prescribe inappropriately for some categories of case and/or be framed in such high-level language as to give little practical guidance. There is not, however, a binary choice between a uniform interpretation of a sufficient interest or characterising standing as ‘context-sensitive’ and saying nothing more. What the appellate courts could usefully undertake is the sort of exercise undertaken in this article: namely, that of dissecting different *types* of judicial review case and offering guidance on how to determine what constitutes a sufficient interest in each.

Thirdly, the analysis above highlights that there is no singular, straightforward answer to the question ‘What is standing for?’ Academic literature on standing has tended to posit two opposing options: standing’s purpose is either to identify whether the applicant has a private right or interest of the kind protected in judicial review, or to ensure that applications are brought only by those well placed to represent the public interest.^215^ These answers, in turn, are derived from two influential, theoretical ‘models’^216^ of judicial review which Varuhas terms the ‘rights’ and ‘public interest’ conceptions.^217^ This article has approached things differently. Rather than working top-down from a theoretical account of judicial review, it has worked upwards from the full range of decided case law. Adopting this approach shows that standing’s purposes are not singular but multiple, and that the requirement to show a ‘sufficient interest’ is performing different roles in different cases. In challenges to unfavourable targeted decisions, for instance, standing plays an important role in protecting the subject of a decision from the impacts of unwanted litigation. In contrast, where a non-targeted decision is in issue, the sufficient interest test facilitates broad access to the courts, while safeguarding against excessive costs implications for public authorities and the unnecessary politicisation of judicial review.^218^

## 8. Conclusion

The recent case law discussed throughout this article, despite its importance, has not garnered much academic attention. A major reason for this may be that it primarily constitutes High Court decisions. It is, however, possible to imagine a narrative which may come to be told about it.^219^ The narrative goes like this: judicial review has been under a great deal of political pressure in recent years. Among other things, the UK government has commissioned a broad review of,^220^ and has proposed and enacted^221^ legislative measures limiting, judicial review. All of this has created a hostile environment for courts and is leading to a judicial climb-down,^222^ of which the recent case law on standing is a part. It sees the courts ‘tightening’ up^223^ on their previously liberal approach to standing in an attempt to appease governmental worries about judicial overreach.

This article has deliberately framed the issues very differently. Not only does this narrative depend on speculation about the mindset of judges; it also tells far too simple a story about the evolution of standing. The direction of the case law has never been a straightforward one of courts consistently opening up the gates of judicial review wider and wider.^224^ The context of a case has always mattered.^225^ Recent decisions, such as *DSD*,^226^*GLP, Public First*^227^ and *GLP & Runnymede*,^228^ in which the courts have interpreted the sufficient interest, are not, therefore, well understood as a straightforward U-turn from older jurisprudence. They are better seen as cases raising legal complexities around standing which have always been there but which the courts have not often had the opportunity to confront. Above all else, they highlight the need for serious study of the ways in which courts adapt the standing test to context.

This article has offered such a study. Its central argument is that distinguishing between three different types of challenge—unfavourable targeted, favourable targeted and non-targeted decisions—sheds light on the case law and the different standing issues which are raised across it. Greater attentiveness to this trichotomy in the future would help the courts to develop a richer and more transparent understanding of what it means to say that what constitutes standing to seek judicial review depends on the context.

## Footnotes

1
*AXA General Insurance Ltd, Petitioners* [2011] UKSC 46, [2012] 1 AC 868, [170].

2 ibid [170].

3 As Lord Steyn famously said in *R (on the application of Daly) v Secretary of State for the Home Department* [2001] UKHL 26, [2001] 2 AC 532, [28].

4 See discussion in section 2.

5 eg Town and Country Planning Act 1990, s 288.

6 See eg *Walton v Scottish Ministers* [2012] UKSC 44, [2013] PTSR 51, [96]; *Aireborough Neighbourhood Development Forum v Leeds City Council* [2020] EWHC 45 (Admin).

7 Human Rights Act 1998, s 7; *Re Northern Ireland Human Rights Commission’s Application for Judicial Review* [2018] UKSC 27, [2019] 1 All ER 173; *In the matter of an application by Ewart for judicial review* [2019] NIQB 88.

8 See eg *R (on the application of EU Lotto Ltd) v Secretary of State for Digital, Culture, Media and Sport* [2018] EWHC 3111 (Admin).

9 See eg *Aireborough Neighbourhood Development Forum* (n 6).

10 On the importance of first instance case law generally see Joanna Bell and Elizabeth Fisher, ‘Exploring a Year of Administrative Law Adjudication in the Administrative Court’ [2021] PL 505; Richard Kirkham and Elizabeth O’Loughlin, ‘Judicial Review and Ombuds: A Systematic Analysis’ [2020] PL 680; Joe Tomlinson, ‘Constitutional Rights and Constitutional Design: Moral and Empirical Reasoning (Publication Review)’ [2020] PL 388.

11
*R v Inland Revenue Commissioners, ex parte National Federation of Self-Employed and Small Businesses Ltd* [1982] AC 617 (HL) (*Fleet Street Casuals*).

12
*R v Inspectorate of Pollution, ex parte Greenpeace Ltd (No 2)* [1994] 4 All ER 329.

13
*R v Secretary of State for Foreign and Commonwealth Affairs, ex parte Rees-Mogg* [1994] QB 522 (DC).

14
*R v Secretary of State for Foreign and Commonwealth Affairs, ex parte World Development Movement Ltd* [1995] 1 WLR 386 (QB).

15 Elizabeth Fisher and Sidney Shapiro, *Administrative Competence: Reimagining Administrative Law* (CUP 2020); Elizabeth Fisher, ‘Administrative Tribunals: An Essay about the Legal Imagination of Administrative Law Scholars’ in James Goudkamp, Mark Lunney and Leighton McDonald (eds), *Taking Law Seriously* (Bloomsbury 2022); Joanna Bell and Sarah Nason, ‘Judicial Review Scholarship and the Importance of Expanding Legal Scholarly Imagination’ in Carol Harlow (ed), *A Research Agenda for Administrative Law* (Elgar 2023).

16 See discussion in section 2 below.

17 Senior Courts Act 1981, s 31(3).

18 Law Commission, *Report on Remedies in Administrative Law* (Law Com No 73, 1976).

19 ibid 21–2.

20
*AXA* (n 1) [63]; *Walton* (n 6) [91].

21 See generally John McGarry, ‘The Importance of an Expansive Test of Standing’ (2014) 19(1) JR 60. Examples include *R v HM Treasury, ex parte Smedley* [1985] QB 657 (CA); *Greenpeace* (n 12); *Rees-Mogg* (n 13).

22
*Fleet Street Casuals* (n 11) 644; *World Development Movement Ltd* (n 14) 395.

23
*R v Somerset County Council, ex parte Dixon* [1998] Env LR 111 (QB), 121; *AXA* (n 1) [61] and [170].

24
*Watkins v Aged Seamen’s Homes* [2018] EWHC 2410 (Admin), [18].

25
*R (on the application of O) v Secretary of State for International Development* [2015] EWHC 2371 (Admin), [27].

26
*R (on the application of DSD) v Parole Board* [2018] EWHC 694 (Admin), [2018] 3 WLR 829, [110].

27 Bell and Fisher (n 10) 519.

28
*R (on the application of Cart) v Upper Tribunal* [2011] UKSC 28, [2012] 1 AC 663, [19] and [33].

29 Carol Harlow, ‘Public Law and Popular Justice’ (2002) 65(1) MLR 1.

30 David Feldman, ‘Public Interest Litigation and Constitutional Theory in Comparative Perspective’ (1992) 55(1) MLR 44.

31 Notable exceptions include Matthew Groves, ‘The Evolution and Reform of Standing in Australian Administrative Law’ (2016) 44 FL Rev 167 (writing in the Australian context); Farah Ahmed and Adam Perry, ‘Standing and Civic Virtue’ (2018) 134 LQR 239.

32
*AXA* (n 1); *Walton* (n 6). Notably, both cases originated in Scotland and concerned standing tests other than the ‘sufficient interest’ test.

33 Joanna Bell, *The Anatomy of Administrative Law* (Hart Publishing 2020) ch 7.

34 See eg the Independent Review of Administrative Law (March 2021) (IRAL), 91: ‘as a result of this approach … standing is rarely an issue in the modern law of judicial review—though perhaps it should be’.

35 In particular, the lack of routine public access to High Court permission decisions would be a major obstacle. On the limitations of the informational landscape generally, see eg Mikolaj Barczentewicz, ‘Cart Challenges, Empirical Methods and Effectiveness of Judicial Review’ (2021) 84(6) MLR 1360.

36 For notable examples of this, see *Smedley* (n 21); *Rees Mogg* (n 13).

37 See eg *R (on the application of the Good Law Project) v Secretary of State for Health and Social Care* [2022] EWHC 2468 (TCC) (*GLP, Abingdon*), in which standing occupies over 50 paragraphs of the judgment.

38 IRAL (n 34) 94.

39 Konrad Schiemann, ‘*Locus standi*’ [1990] PL 342, 345.

40 Joe Tomlinson, ‘Crowdfunding and Public Interest Judicial Review: A Risky New Resource for Law Reform’ [2019] PL 166; Sam Guy, ‘Mobilising the Market: An Empirical Analysis of Crowdfunding for Judicial Review Litigation’ (2022) 86(2) MLR 331.

41 Jolyon Maugham, *Bringing Down Goliath: How Good Law Can Topple the Powerful* (Virgin Digital, 2023) 269.

42 <https://goodlawproject.org/about/> accessed 15 August 2023.

43 eg Jonathan Auburn, Jonathan Moffett and Andrew Sharland, *Judicial Review: Principles and Procedure* (OUP 2013) identifies the following factors: ‘the merits of the claim, the existence of better placed challengers, and the nature and reputation of the individual or organisation in question’.

44 eg *GLP, Abingdon* (n 37) [502], where Waksman J identifies six factors: ‘(1) the merit of the underlying claims; (2) the particular legislative or other context of the claim being made; (3) how, if at all, the claimant might be affected by the unlawfulness alleged; (4) the gravity of the allegations or findings made; (5) other possible claimants; and (6) the position of the actual claimant’.

45 As highlighted in Paul Craig, ‘Taxonomy and Public Law: A Response’ [2019] PL 281, 292.

46 eg Varda Bondy, Lucinda Platt and Maurice Sunkin, *The Value and Effects of Judicial Review* (Public Law Project, 2015) 22–4, where, across a total of 502 cases, the authors classified 75% as ‘own fact’ cases. See also Bell and Fisher (n 10) 519; Sarah Nason, *Reconstructing Judicial Review* (Hart Publishing 2017) ch 4.

47 The distinction overlaps but is different to that drawn by Megarry VC in *McInnes v Onslow-Fane* [1978] 1 WLR 1520 (Ch) between ‘forfeiture’ and ‘application’ cases, in that unfavourable untargeted decisions include denials of benefits not presently enjoyed.

48 See eg *Professional Standards Authority for Health and Social Care v General Medical Council (Battah)* [2022] EWHC 2075 (Admin).

49 The Financial Restrictions (Iran) Order 2009, the subject of the challenge in *Bank Mellat v HM Treasury* [2013] UKSC 39, [2014] AC 700, is an unusual example of a statutory instrument which was ‘targeted’ at two named banks.

50
*R (on the application of Miller) v Secretary of State for Exiting the European Union* [2016] EWHC 2768 (Admin) (note the discussion of standing at [7]).

51
*R (on the application of Hutchinson) v Secretary of State for Health and Social Care* [2018] EWHC 1698 (Admin) (note the discussion of standing at [138]–[147]).

52 Civil Procedure Rules 1998, Part 54, rule 1.

53 eg *Hertfordshire County Council v Secretary of State for Housing, Communities and Local Government* [2021] EWHC 1093 (Admin), [2021] 1 WLR 3714. Such cases are, however, unusual and raise questions concerning whether the court should exceptionally depart from the usual starting point that academic issues cannot be raised in judicial review (see eg *Devon Partnership NHS Trust v Secretary of State for Health and Social Care* [2021] EWHC 101 (Admin), [2021] 1 WLR 2945, [11]–[12]).

54 Note, however, that there may be specific grounds the subject of the decision lacks standing to raise. An example is *R (on the application of Devonhurst) v Luton Borough Council* [2023] EWHC 978 (Admin), in which a developer who had standing to challenge an enforcement notice requiring demolition of their property could not rely on an alleged breach of the rights of residents conferred by Article 8 of the European Convention on Human Rights.

55 Craig (n 45) 292.

56 Court of Protection Rules 2017, s 1; *Practice Direction 17A—Litigation Friend.*

57 Peter Cane, ‘Standing up for the Public’ [1995] PL 276, 276.

58 See also the comment of Singh LJ and Swift J in *R (on the application of Good Law Project & Runnymede Trust) v Prime Minister* [2022] EWHC 298 (Admin) (*GLP, Runnymede*) that ‘if the facts of *Ridge v Baldwin* were to occur today but the Chief Constable did not bring proceedings himself … it is very difficult to see how an NGO could bring those proceedings’. Similar remarks were made by the Privy Council in the older case *Durayappah v Fernando* [1967] 2 AC 337 (PC), 353.

59
*R (on the application of Zafar) v Secretary of State for Levelling Up, Housing and Communities* [2022] EWHC 2154 (Admin).

60
*R (on the application of AB) v A County Council* [2022] EWHC 2707 (Admin), [2023] ACD 11.

61
*Re Conway & Hutchinson* [2011] NIQB 68.

62
*R v Legal Aid Board, ex parte Bateman* [1992] 1 WLR 7111.

63
*Re Moon’s Application for leave to apply for Judicial Review* [2021] NIQB 26. Note that clients have brought successful challenges against ‘non-targeted’ measures concerning the remuneration of legal representatives. See eg *In the matter of an application of Raymond Brownlee for Judicial Review* [2014] UKSC 4, in which the UK Supreme Court found that the Legal Aid for Crown Court Proceedings (Costs) Rules (Northern Ireland) 2005 were *ultra vires*.

64 Although the High Court queried whether the Company’s Objects permitted it initiate judicial review: *Re JR216’s Application for Judicial Review* [2022] NIKB 28, [89]–[93].

65 ibid [76]–[77] (although, as discussed below, the latter had standing to challenge the ombudsman’s practice more broadly ([78]–[79] and [89]–[93]).

66 Walton (n 6) [88].

67
*R v Birmingham City Council, ex parte Connolly* (1994) 26 HLR 551.

68 DSD (n 26) [110]; Bell, *The Anatomy of Administrative Law* (n 33) ch 7.

69
*Zafar* (n 59) 16.

70
*R (on the application of the Law Society) v Legal Services Commission* [2007] EWHC 1848 (Admin), [112]. See generally *R (on the application of Privacy International) v Investigatory Powers Tribunal* [2019] UKSC 22, [2020] AC 491.

71
*AB* (n 60) [6].

72 Defined at Civil Procedure Rules 1998, rule 54.1(f).

73 See generally Tom Hickman, ‘Public Law’s Disgrace’ (*UK Constitutional Law Blog*, 9 Feb 2017) <https://ukconstitutionallaw.org/>.

74 See eg *R (on the application of Easter) v Mid-Suffolk District Council* [2020] EWCA Civ 1378, in which a developer appearing as an interested party was held liable for costs of £19,338.84 in respect of a judicial review challenge to a grant of planning permission.

75 See discussion in section 5 below.

76 See generally discussion in *R (on the application of Good Law Project) v Secretary of State for Health and Social Care* [2021] EWHC 346 (Admin) (*GLP, transparency*), [96]–[97].

77 Hickman (n 73); Tomlinson (n 40).

78 See generally discussion in Jo Miles, ‘Standing under the Human Rights Act 1998: Theories of Rights Enforcement and the Nature of Public Law Adjudication’ (2000) 59(1) CLJ 133.

79
*R (on the application of Islam) v Secretary of State for the Home Department* [2019] EWHC 2169 (Admin), [2019] ACD 131.

80 On the requirement of giving notice of citizen deprivation decisions, see *R (on the application of D4) v Secretary of State for the Home Department* [2021] EWHC 2179 (Admin), [2021] ACD 110.

81
*Islam* (n 79) [21].

82
*DSD* (n 26) [10].

83 ibid.

84
*R (on the application of McCourt) v Parole Board* [2020] EWHC 2320 (Admin), [2020] ACD 127, [41] (emphasis added).

85 For example, on standing to challenge individual employment decisions, see *GLP, Runnymede* (n 58); on standing to challenge grants from the foreign aid fund, see *World Development Movement* (n 14); *O* (n 25).

86 Town and Country Planning Act 1998 (TCPA), s 57.

87 TCPA, s 77.

88 TCPA, s 78.

89 The Town and Country Planning (Development Management Procedure) (England) Order 2010 (SI 2010/2184), reg 13 and Sch 3.

90 See eg Planning Inspectorate, *Guide to Taking Part in Planning and Listed Building Consent Appeals Proceeding by Written Representations—England* (April 2023). Similar guidance exists in relation to appeals proceedings by hearing and by inquiry.

91 TCPA, s 288.

92 For examples of cases in which applicants lacked a sufficient interest due to insufficient participation, see *Morbaine Ltd v First Secretary of State* [2004] EWHC 1708 (Admin), [2005] JPL 377; *Eco-Energy Ltd v First Secretary of State* [2004] EWCA Civ 1566, [2005] 2 P & CR 5; *Ashton v Secretary of State for Communities and Local Government* [2010] EWCA Civ 600, [2011] 1 P & CR 5.

93
*Turner v Secretary of State for the Environment* (1974) 28 P & CR 123 (QB), 139. See also *Walton* (n 6) [86].

94
*Lardner v Renfrewshire District Council* 1997 SC 104, 108.

95 ibid 108. See eg *Cumming v Secretary of State for Scotland* 1992 SC 463.

96 See also the unusual case of *Duff v Causeway Coast and Glens Borough Council* [2023] NICA 22.

97
*Lardner* (n 94) 107.

98 See eg the older case *Gregory v Camden LBC* [1966] 1 WLR 899 (QB) (concerning standing to seek a declaration).

99 Although on the practical difficulties of participating in the planning process, see eg Carolyn Abbot, ‘Losing the Local? Public Participation and Legal Expertise in Planning Law’ (2020) 40(2) LS 269; Chiara Armeni, ‘Participation in Environmental Decision-making: Reflecting on Planning and Community Benefits for Major Wind Farms’ (2016) 28(3) JEL 415.

100 See section 3.

101
*DSD* (n 26) [113]–[114].

102
*McCourt* (n 84).

103
*DSD* (n 26) [109]–[112].

104
*McCourt* (n 84) [50].

105 ibid [44].

106 The analogy is imperfect, however, both because the Attorney General and the General Medical Council are public authorities, and because their roles are the consequence of specific legislation, rather than judicial interpretation of the ‘sufficient interest’ test.

107 See *R (Bulger) v Secretary of State for the Home Department* [2001] EWHC 119 (Admin); *McCourt* (n 84) [47]–[48].

108 Medical Act 1983, s 40A. (Note, however, ongoing proposals to repeal this provision.)

109 As illustrated, for instance, by the recent case *R (on the application of Bailey) v Secretary of State for Justice* [2023] EWHC 555 (Admin).

110 Parole Board Rules 2019 (SI 2019/1038) (PBRs), rule 28 (as amended).

111 It is noteworthy that, following recent reforms, victims may ask the SSJ to request reconsideration: see Ministry of Justice, ‘Requesting Reconsideration of a Parole Board Decision to Release a Prisoner’ (13 March 2020).

112
*DSD* (n 26) concerned a challenge to the Parole Board’s long-standing understanding of its own legal duties as including a requirement to place no weight on unproven allegations. See now generally *R (on the application of Pearce) v Parole Board* [2023] UKSC 13.

113 See introductory text to section 4.

114
*McCourt* (n 84) [50].

115 Public Contract Regulations 2015 (SI 2015/102) (PCRs). Note that some of the decided case law concerns earlier iterations of these regulations. Note also that the regulations have now been replaced by the Procurement Act 2023.

116 PCRs, reg 5. See now Procurement Act 2023, Sch 1.

117 PCRs, regs 26–32. See now Procurement Act 2023, Part 3.

118 PCRs, reg 18. See now Procurement Act 2023, s 12.

119 See eg *R (on the application of Good Law Project Ltd & Everydoctor) v Secretary of State for Health and Social Care* [2022] EWHC 46 (TCC) (in which the grounds of challenge included the inadequacy of reasons and irrationality).

120 PCRs, regs 89–90.

121 PCRs, reg 91. See now Procurement Act 2023, s 100.

122
*R (on the application of Chandler) v Secretary of State for Children, Schools and Families* [2009] EWCA 1011, [77].

123 ibid.

124 At the time, the Public Contracts Regulations 2006 (SI 2006/5) were in force.

125
*Chandler* (n 122) [77].

126 This is not to say that there is no room for debate about the outer margins. For example, one question which has arisen in the planning case law is whether an individual has standing to review where they were part of a group who participated in decision making. In *Ashton* (n 93), the Court of Appeal held this to be insufficient participation.

127 Another concerns the question of whether *Chandler* is to be understood as having established two categories of standing or whether the overarching test is one of motive: see eg *R (on the application of Gottlieb) v Winchester City Council* [2005] EWHC 231 (Admin), [2015] ACD 74, [153].

128
*R (on the application of Wylde) v Waverley BC* [2017] EWHC 466 (Admin), [37].

129
*GLP, transparency* (n 76) [94].

130 See generally Joanna Bell, ‘Remedies in Judicial Review: Confronting an Intellectual Blindspot’ [2022] PL 200, 210–11.

131 eg *R (on the application of London Secure Services Ltd) v Youth Justice Board* [2009] EWHC 2347 (Admin); *AJ v Calderdale Borough Council* [2012] EWHC 3552 (Admin).

132
*Chandler* (n 122) [36].

133 See discussion in *R (UNISON) v NHS Wiltshire* [2012] EWHC 624 (Admin), [10].

134
*Chandler* (n 122) [72]. It is worth noting that this criticism applies to other instances in which the courts have relied on ‘gravity’ as a factor in assessing the sufficiency of an applicant’s interest. See eg *Fleet Street Casuals* (n 14) 618; *World Development Movement* (n 11) 395.

135 Contrast the opposing views of O’Farrell in *R (on the application of Good Law Project) v Minister for the Cabinet Office* [2021] EWHC 1569 (Admin), [181] (an appeal against this decision has since been allowed on different grounds) and Waksman J in *GLP, Abingdon* (n 37) [536].

136
*R (on the application of the Good Law Project) v Minister for the Cabinet Office* [2022] EWCA Civ 21, [2022] PTSR 933 (*GLP, Public First*).

137 ibid [6].

138 See eg Procurement Act 2023, s 12.

139 The exclusive parties to hearings are the SSJ and the prisoner (PBRs, rule 2). Victims, although their participatory rights have recently been extended (Ministry of Justice, *Code of Practice for Victims of Crime in England and Wales (Victim’s Code)* (April 2021), play a highly restricted role and hearings are closed to the public (PBRs, rule 15).

140 Procurement operates as an essential private process, with no mandatory public consultation.

141
*McCourt* (n 84) [41].

142 Human Rights Act 1998, s 6.

143 Equality Act 2010, s 149.

144 Ivan Hare and others, *De Smith’s Judicial Review of Administrative Action* (9th edn, Sweet & Maxwell 2023).

145 Bell, *The Anatomy of Administrative Law* (n 33) ch 3.

146 See generally *R (on the application of UNISON) v Lord Chancellor* [2017] UKSC 51, [67]–[72].

147
*Cart* (n 28) [19] and [33].

148 Perhaps subject to the requirement that they are not acting for an ill motive of the kind that might threaten the integrity of judicial review. See eg *Feakins v Department for the Environment, Food and Rural Affairs* [2006] EWCA Civ 699.

149 It is noteworthy in particular that many procurement decisions are subject to a 30-day time limit: see CPRs, Part 54, rule 5.

150
*McCourt* (n 84) [41].

151
*Gregory* (n 98).

152 Town and Country Planning (Environmental Impact Assessment) Regulations 2017 (SI 2017/571). See also Levelling-up and Regeneration Act 2023, Part 5.

153 Equality Act 2010, s 149.

154 Note, however, that the question may arise of whether an applicant is in reality seeking to complain about a general practice or a series of individual targeted decisions. See eg *GLP, Runnymede* (n 58).

155 Notable examples include *R (on the application of Howard League for Penal Reform) v Lord Chancellor* [2017] EWCA Civ 244; *R (on the application of Huxtable) v Secretary of State for Justice* [2021] EWCA Civ 1394, [2022] 1 WLR 813; *R (on the application of Bailey) v Secretary of State for Justice* [2023] EWHC 555 (Admin), [2023] ACD 59.

156 See eg *GLP, transparency* (n 76) [102].

157 ibid [96].

158
*JR216* (n 64) [79].

159 ‘Individual’ is used here in a broad sense, to encompass both natural and legal persons.

160 These are not exhaustive possibilities. For instance, a significant portion of judicial review challenges are brought by public authorities. Standing issues have not, however, often arisen in such cases. Notable exceptions are *DSD* (n 26); *R (on the application of Doncaster Metropolitan Borough Council) v Doncaster Sheffield Airport Ltd* [2022] EWHC 3060 (Admin).

161 Cane (n 57).

162 There are many examples of challenges of this kind in which the standing of the organisation has not been contested. Recent notable examples include *R (on the application of United Trade Action Group Ltd) v Transport for London* [2021] EWCA Civ 1197; *R (on the application of Associated Society of Locomotive Engineers and Firemen) v Secretary of State for Business and Trade* [2023] EWHC 1781 (Admin).

163
*JR216* (n 64) [78].

164
*R (on the application of Adiatu) v HM Treasury* [2020] EWHC 1554 (Admin), [2021] 2 All ER 484, [136].

165 Cane (n 57) 279.

166 ibid 278.

167 Note that, as Cane highlights (ibid 281), Otton J’s reasoning is multifaceted.

168
*Greenpeace* (n 12) [70].

169
*GLP, transparency* (n 76) [104].

170 ibid.

171
*GLP, Abingdon* (n 37) [541].

172
*GLP, Runnymede* (n 58) [21] (giving the example of *R (on the application of Motherhood Plan) v HM Treasury* [2021] EWCA Civ 1703).

173 eg *R (on the application of ClientEarth) v Secretary of State for Environment, Food and Rural Affairs* [2015] UKSC 28, [2015] 4 All ER 724.

174 eg *R (on the application of ClientEarth) v Secretary of State for Business, Energy and Industrial Strategy* [2020] EWHC 1303 (Admin).

175 <www.clientearth.org/> accessed 31 August 2023.

176 See eg *Bristol Airport Action Network Coordinating Committee v Secretary of State for Levelling Up, Housing and Communities* [2023] EWHC 171 (Admin).

177 Note that the applicants’ claim to also be challenging a general practice was rejected by the court: *GLP, Runnymede* (n 58) especially [95].

178
*World Development Movement* (n 14) (cited as an example at *GLP, Runnymede* (n 58) [21]).

179
*R (on the application of Refugee Legal Centre) v Secretary of State for the Home Department* [2004] EWCA Civ 1481 (cited as an example at *GLP, Runnymede* (n 58) [21]).

180 Perhaps a reflection of its history. See Maugham (n 41); *GLP, Runnymede* (n 58) [55]–[56].

181
*GLP, Runnymede* (n 58) [56].

182 ibid [57].

183 The GLP has reported of a permission decision in which the High Court rejected the contention that the effect of *GLP, Runnymede* is that it ‘will never have standing’ <https://goodlawproject.org/update/permission-denied-children-care/> accessed 31 August 2023.

184
*GLP, transparency* (n 76).

185 eg *Miller* (n 50); *R (on the application of Friends of the Earth) v Secretary of State for Business, Energy and Industrial Strategy* [2022] EWHC 1841 (Admin).

186
*GLP, Runnymede* (n 58) [21].

187 eg the practices challenged in *GLP, transparency* (n 76).

188 eg *Malloch v Aberdeen Corporation* [1971] 1 WLR 1578; *R (on the application of Shoesmith) v Ofsted* [2011] EWCA Civ 642, [2011] I.CR 1995.

189 See discussion in section 5 above.

190 Note the Divisional Court did recognise an exception for challenges to alleged breaches of the public sector equality duty. The Runnymede Trust was found to have standing to raise this issue in judicial review: *GLP, Runnymede* (n 58) [59].

191 Note that organisations have also established standing by identifying such an impact. Examples include *DSD* (n 26) (where News Group Newspapers Ltd had standing to challenge a rule in the PBRs which inhibited the ability of journalists to investigate and report on parole decisions).

192
*AXA* (n 1) [63].

193
*Royal Cayman Islands Police Association v Commissioners of the Royal Cayman Islands Police Service* [2021] UKPC 21.

194 eg *R (on the application of DM) v Secretary of State for the Home Department* [2023] EWHC 740 (Admin).

195
*Adiatu* (n 164).

196 Contrast the position taken in *Gregory* (n 98) on standing to seek a declaration before the procedural reforms.

197 Contrast the view of Schiemann J in *R v Secretary of State for the Environment, ex parte Rose Theatre* [1990] 1 QB 504 (QB), 522.

198
*Walton* (n 6) [94].

199 See also *Hutchinson* (n 51).

200
*Miller* (n 50) [7].

201
*R (on the application of Jones) v Commissioner of Police of the Metropolis* [2019] EWHC 2957 (Admin), [2020] 1 WLR 519, [61].

202
*R (on the application of Peters) v Secretary of State for Health and Social Care* [2021] EWHC 3182 (Admin), [34].

203 Note the complexity discussed in *DM* (n 194) of whether the alleged ongoing failure constituted a ‘function’.

204
*JS v Secretary of State for the Home Department* [2021] EWHC 234 (Admin).

205
*Fleet Street Casuals* (n 14) 644.

206 See Law Commission, *Administrative Law: Judicial Review and Statutory Appeals* (Law Com No 226, 1994) and criticisms in Harlow (n 29).

207
*Jones* (n 201) [62].

208
*GLP, transparency* (n 76) [107]: ‘No doubt, the addition of politicians as parties may raise the profile of the litigation. It may make it easier to raise funds. But these are not proper reasons for adding parties.’

209 It should also be noted that the UK Supreme Court in *Walton* (n 6) emphasised the importance of not precluding ‘individuals who are sufficiently concerned, and sufficiently well-informed’ from contesting alleged breaches of environmental law’ ([153]).

210 See generally Elizabeth Fisher and Jeremy Kirk, ‘Still standing: An Argument for Open Standing in Australia and England’ (1997) 71(5) ALJ 370.

211 eg *Islam* (n 79).

212
*JR216* (n 64) [79].

213
*AXA* (n 1) [62].

214 See eg Joanna Bell, ‘Embracing the Unwanted Guests at the Judicial Review party: Why Administrative Law Scholars Should Take Planning Law Seriously’ in Maria Lee and Carolyn Abbott (eds), *Taking Planning Law Scholarship Seriously* (UCL Press 2022).

215 See eg Mark Elliott and Jason Varuhas, *Administrative Law* (5th edn, OUP 2017) ch 14.

216 For a critique see Bell, *The Anatomy of Administrative Law* (n 33).

217 Jason Varuhas, ‘The Public Interest Conception of Public Law: Its Procedural Origins and Substantive Implications’ in John Bell and others (eds), *Public Law Adjudication in Common Law Systems* (Hart Publishing 2016).

218 See especially *Jones* (n 201), discussed above.

219 See eg <https://goodlawproject.org/public-enemy-number-one/> accessed 31 August 2023.

220 IRAL (n 34).

221 eg Judicial Review and Courts Act 2022, s 2; Dissolution and Calling of Parliament Act 2022, s 3; Illegal Migration Act 2023, s 13(4).

222 On culture within the UK Supreme Court, see eg Conor Gearty, ‘In the Shallow End’ (2022) 44(2) London Review of Books; Lewis Graham, ‘The Reed Court by Numbers: How Shallow Is the Shallow End’ (*UK Constitutional Law Blog*, 4 April 2023) <https://ukconstitutionallaw.org>.

223
*Duff* (n 96) [26].

224 Bell (n 33) ch 7.

225 See eg the judgment of Lord Wilberforce in *Fleet Street Casuals* (n 14).

226
*DSD* (n 26).

227
*GLP, Public First* (n 136).

228
*GLP, Runnymede* (n 58).

